# Dual-Laser PBF-LB Processing of a High-Performance Maraging Tool Steel FeNiCoMoVTiAl

**DOI:** 10.3390/ma14154251

**Published:** 2021-07-29

**Authors:** Gregor Graf, Niki Nouri, Stefan Dietrich, Frederik Zanger, Volker Schulze

**Affiliations:** 1 Rosswag GmbH, 76327 Pfinztal, Germany; 2 Institute for Applied Materials-Materials Science and Engineering (IAM-WK), Karlsruhe Institute of Technology (KIT), 76131 Karlsruhe, Germany; niki.nouri@kit.edu (N.N.); stefan.dietrich@kit.edu (S.D.); volker.schulze@kit.edu (V.S.); 3 wbk Institute of Production Science, Karlsruhe Institute of Technology (KIT), 76131 Karlsruhe, Germany; frederik.zanger@kit.edu

**Keywords:** laser powder bed fusion, process development, maraging tool steel, FeNiCoMoVTiAl, Specialis, parameter studies, dual-laser PBF-LB, multi-laser PBF-LB, mechanical characterization, high hardness, functionally graded

## Abstract

As part of an international research project (HiPTSLAM), the development and holistic processing of high-performance tool steels for AM is a promising topic regarding the acceptance of the laser powder bed fusion (PBF-LB) technology for functionally optimized die, forming and cutting tools. In a previous work, the newly developed maraging tool steel FeNiCoMoVTiAl was qualified to be processed by laser powder bed fusion (PBF-LB) with a material density of more than 99.9% using a suitable parameter set. To exploit further optimization potential, the influence of dual-laser processing strategies on the material structure and the resulting mechanical properties was investigated. After an initial calibration procedure, the build data were modified so that both lasers could be aligned to the same scanning track with a defined offset. A variation of the laser-based post-heating parameters enabled specific in-situ modifications of the thermal gradients compared to standard single-laser scanning strategies, leading to corresponding property changes in the produced material structure. An increase in microhardness of up to 15% was thus obtained from 411 HV up to 471 HV. The results of the investigation can be used to derive cross-material optimization potential to produce functionally graded high-performance components on PBF-LB systems with synchronized multi-laser technology.

## 1. Introduction

The industrial usage of metal additive manufacturing (AM) is steadily increasing in many branches and applications. For the tooling industry, the integration of internal channel structures enables new design possibilities with increased performance due to better heat transfer for die, forming and cutting applications [1,2,3]. The most common AM technology for high-quality metal parts is the laser powder bed fusion (PBF-LB) process [4]. The PBF-LB process enables functional benefits to the part with a higher freedom of design due to the layerwise manufacturing with metal powder as the starting material and a laser beam as the energy source for melting. The resulting material properties differ from conventional manufacturing routes due to the local micro-welding processes. The solidification after melting is characterized by high cooling rates (10^3^–10^8^ K/s) based on self-quenching by solidified previous layers and thus leading to fine-grained material microstructures [5,6].

### 1.1. High-Performance Tool Steels for PBF-LB

High-performance tool steels with emphasis on high hardness often contain high amounts of carbon (≥0.4 wt%), resulting in a poor weldability due to the increased hot cracking sensitivity. The PBF-LB processing of high-carbon steels with industrial standard PBF-LB systems and preheating temperatures of 200 °C or less thus often leads to cracking issues due to the high local stresses associated with the volumetric change during phase transformation as well as the repetitive heating and cooling cycles [7,8,9,10,11].

Maraging tool steels are high alloyed steels with more than 12 wt% nickel and known for their good mechanical properties, with a combination of high strength and toughness as well as high temperature strength, which leads to further potential for their function-optimized usage in the AM tooling industry [12]. Due to the low amount of carbon, with less than 0.03 wt%, the necessary weldability for PBF-LB processes is given. The optimization of the mechanical properties is based on the precipitation of intermetallic phases during aging in a common temperature range from 480 °C to 500 °C for 2 h to 6 h, which results in significantly higher hardness [13,14]. Furthermore, the possibility of short-term aging of maraging tool steels was demonstrated in a study by Marcisz and Stępień [15] for 18Ni350, leading to an increase in hardness from 340 HV to 600 HV with a temperature of 600 °C applied for only 15 s. The increased hardness was probably due to a redistribution of the atoms before precipitation.

For the additive manufacturing of mechanically loaded tools, the most common material in industrial PBF-LB systems is the maraging tool steel AISI 18Ni300. In the as-printed state, the alloy has an average hardness of around 330 HV, which leads to good machinability. The maximum hardness after heat treatment of 18Ni300 is limited in a range of around 540 HV to 665 HV [13,16,17]. Therefore, the resulting hardness is often not suitable for many applications in the tooling industry. Here, for example, high-speed steels such as the AISI M2 are broadly used with a hardness of more than 700 HV [13].

In a previous study, the newly developed maraging tool steel alloy FeNiCoMoVTiAl was initially qualified within a holistic process chain for use in standard PBF-LB systems [18]. The results showed good PBF-LB processability, with significantly higher hardness compared to the reference material 18Ni300 of nearly 700 HV after heat treatment.

### 1.2. Multi-Laser PBF-LB Processing

The industrial application of multi-laser PBF-LB scanning strategies started with the market readiness of the first commercially available multi-laser PBF-LB machines in 2011 [19]. The accompanying multi-laser scanning strategies were mainly based on stitching the scanning vectors of multiple lasers together in defined overlapping areas to build larger parts more efficiently and enable larger build spaces. The resulting influence of using multiple lasers to jointly build parts is exemplarily shown in [20].

Using multiple lasers on the same scanning track with a predefined offset is a field of research that is relatively underrepresented. There are some early but promising results from Abe et al. [21] that showed that the bending strength and hardness can be modified with a dual-laser scanning strategy through slower cooling or reheating. However, following studies mainly focused on the usage of a second laser beam for preheating the powder bed to reduce melting fumes [22] or to influence the density and surface quality with a very close following second laser beam [23]. Further findings in [24] based on simulations showed the possibility of reducing the thermal gradients by using a second defocused laser beam for post-heating. Other studies used multiple laser beams or laser diode area melting to obtain a higher build rate than the standard PBF-LB process based on the increased amount of energy sources applied simultaneously to the powder bed [25,26].

This paper focuses on the effects of a dual-laser processing strategy with the second laser beam used for in-situ heat treatment by remelting a fraction of the welded tracks with a delay of less than 10 ms. Part of the additional energy introduced by the subsequent laser track heats the surrounding material structure and modifies the thermal history. The resulting melt pool dimensions and hardness will be correlated with the varied process parameter sets to deepen the knowledge about the further application of dual-laser strategies. The presented results could furthermore be used to derive PBF-LB processing strategies for voxel-based graded material structures with maraging tool steels and other materials. Similar results based on modified thermal gradients were shown in [27] with directed energy deposition (DED) processes and a Fe19Ni5Ti steel designed for laser additive manufacturing leading to a Damascus-like metallic composite.

## 2. Experimental Setup

### 2.1. Material

The maraging tool steel FeNiCoMoVTiAl, also known under the trademark Specialis^®^, is based on a newly developed chemical composition, as shown in Table 1. The alloy is designed for good PBF-LB processability and higher hardness after heat treatment compared to other maraging steels. Other advantages of this alloy to be examined could be increased high-temperature strength and high-temperature hardness, which is an essential feature when using this alloy for applications in thermally stressed dies and tools.

The pre-alloyed material was atomized on a small batch atomizer AU 3000 system by BluePower Casting Systems GmbH (Walzbachtal, Germany). Further details about the system and the associated powder production process can be found in [28,29]. The resulting metal powder was processed according to PBF-LB powder specifications for good powder bed density and flowability. The analysis results regarding the particle size distribution, particle sphericity, flowability and residual moisture are shown in Table 2.

### 2.2. Machine

**Figure 1:**
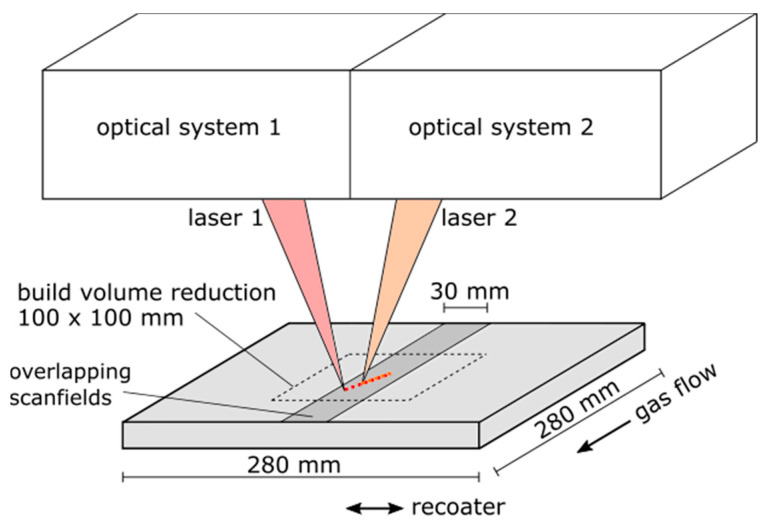
Illustration of the PBF-LB system configuration to achieve dual-laser processing.

The experiments were conducted on the PBF-LB system SLM 280 1.0 Twin 400 W with gas flow upgrade by SLM Solutions Group AG (Lübeck, Germany). The optical system consisted of two water-cooled 400 W fiber lasers of 1070 nm wavelength (YLR-400-WC) by IPG Laser GmbH (Burbach, Germany), with each laser connected to an air-cooled and digitally controlled galvo system with varioSCAN by SCANLAB GmbH (Puchheim, Germany). The two laser systems were calibrated to work separately on their assigned half of the build plate and furthermore using stitching operations to produce larger parts within a predefined overlapping area of 280 mm × 30 mm (see Figure 1). A build volume reduction of 100 mm × 100 mm was used in this study to lower the amount of metal powder needed for a certain build height. To enable the synchronized switching and movement operations of both laser beams within the overlapping area, the two SCANLAB RTC^®^5 control cards were connected for synchronized clock rates in a master–slave mode. According to SCANLAB, a reproducible time lag of around 0.16 µs could be achieved [31]. While the standard machine control software (SLM MCS 2.3 Build 78) with a build processor exported file could not use the synchronization features, a special version of the MCS was provided by SLM Solutions Group AG. With this software update, the parameters for each scanning and jumping vector of the lasers could be defined individually by importing a specific formatted csv-table for a behavior analogous to G-code programming.

### 2.3. Double-Laser Exposure Strategy

**Figure 2:**
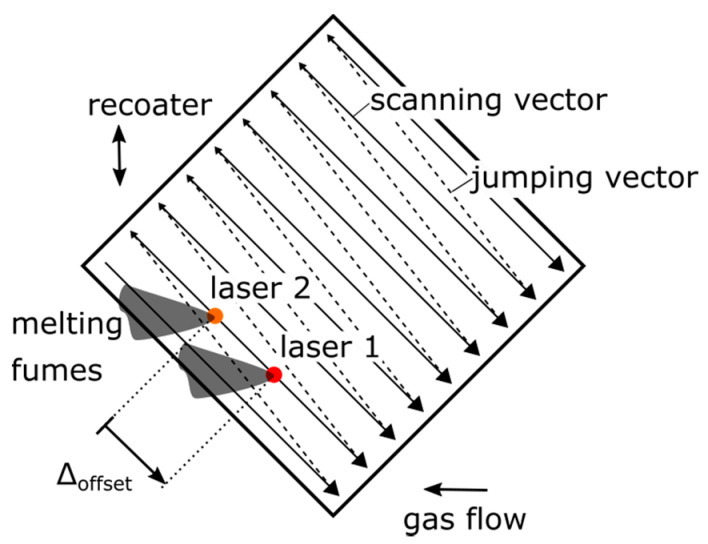
Dual-laser scanning strategy for specimen manufacturing.

The disadvantage of using the modified MCS with csv-file input lies in the inability to change the exposure strategy during the manufacturing process. The initially defined scanning and jumping vectors will be repeated within each layer and the common rotation of the scanning strategy for reducing the resulting material defects and thermally induced residual stresses cannot be implemented. Therefore, an exposure strategy was derived to enable a stable and reproducible manufacturing process to produce volumetric sample parts with two laser beams scanning the same vectors with a predefined offset. The exposure strategy is exemplarily shown in Figure 2. The offset between the two laser beams was regulated by a longer approach path for the start vector of one laser beam and remained constant during manufacturing due to the synchronized clock rates of the optical systems. Furthermore, an orientation angle of 45° regarding the recoater and gas flow direction was determined to eliminate negative impacts by recoating or melting parallel to the scanning vectors. With the shown exposure strategy, the resulting melting fumes of laser 1 should not lead to a negative influence of laser 2 as long as both laser beams are not working with an offset of one or multiple scanning vector lengths.

### 2.4. Calibration Procedure

**Figure 3:**
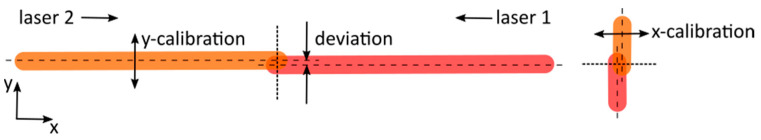
Illustration of the procedure for spatial calibration.

Both optical systems and the associated controlling units of the two lasers must be calibrated to minimize the spatial and temporal deviations. For the spatial calibration, a simple pattern according to Figure 3 was created on multiple positions of a build plate within the reduced overlapping area of 100 mm × 30 mm. The possible deviations for X-axis and Y-axis were measured by microscopy. The optical systems were adjusted within the MCS so that all measured deviations were reproducibly smaller than 5 µm on both axes for a melt pool width of approximately 120 µm.

**Figure 4:**
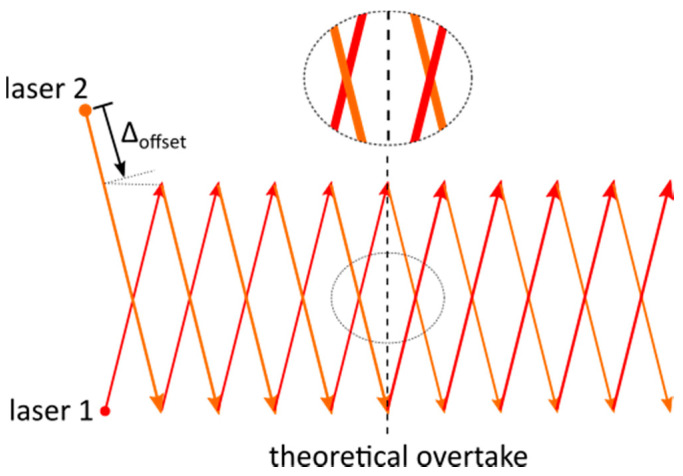
Illustration of procedure for temporal calibration.

The temporal calibration was validated to some extent by creating the intersecting pattern according to Figure 4 on a build plate. Both laser beams were scanning along the respectively associated vectors, with one laser delayed at the start due to an extended vector. This delay will theoretically be compensated after scanning half of the vectors by a faster scanning speed. During analysis of the intersection points via microscopy, the subsequent laser beam could be identified by the overlying melt pool. The theoretical time lag of 0.16 µs was difficult to prove with this calibration approach because of the melting and solidification inertia consequently leading to merging melt pools if the two laser beams were intersecting in close succession. As a result, a reproducible time lag variation of 0.6 ms as a maximum could be derived regarding the used measurement approach. To determine this value, the vector length between the two intersection points with change in overlying melt pool was divided by the scanning velocity. We expected a significantly smaller variation in the time lag than the proven 0.6 ms because of the high reproducibility of measurement results during the test series.

### 2.5. Experimental Design

**Figure 5:**
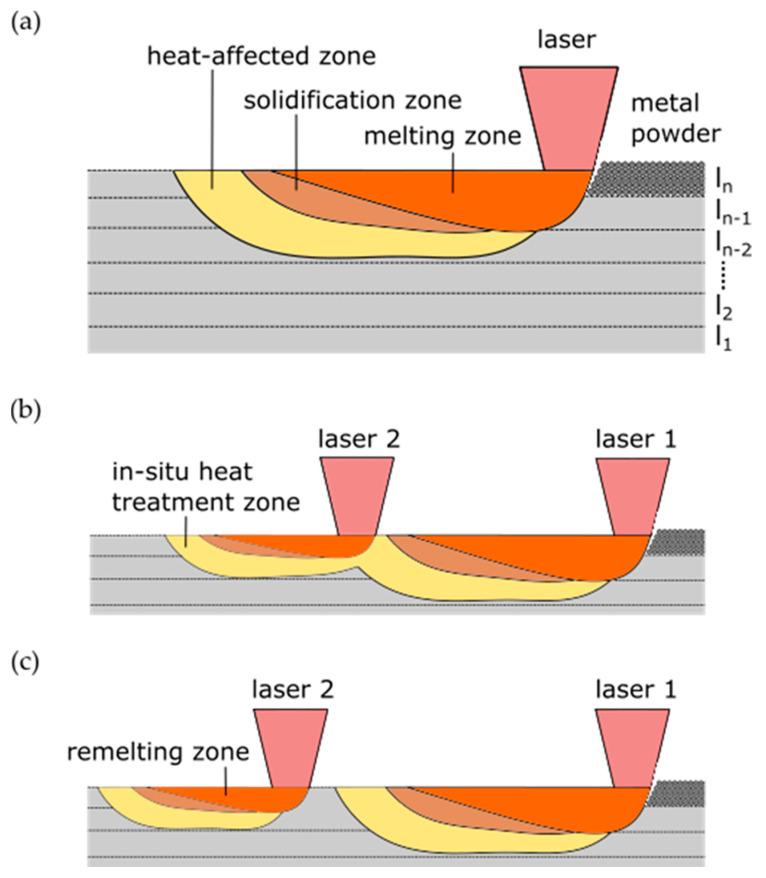
Longitudinal section of the PBF-LB processing with (**a**) single laser, (**b**) dual-laser in-situ heat treatment and (**c**) dual-laser remelting strategy.

To investigate the influence of the dual-laser exposure strategies, a variation in post-heating parameters was used to produce volumetric samples for comparison with a standard single-laser and remelting strategy. In Figure 5, the main characteristics of the three production conditions regarding the energy input and resulting melting, solidification and in-situ heat treatment zones are shown. The elaborated dual-laser exposure strategies shown in Figure 5b were used to extend the time period of in-situ heat treatment compared to the single-laser processing in Figure 5a, with the goal of influencing the resulting mechanical properties of the maraging tool steel. If the offset distance between the two laser beams is substantially extended and the material has already a homogeneous temperature level when the second laser beam is approaching, an approximation of well-known remelting process conditions shown in Figure 5c should be obtained. Due to a less penetrating melt pool depth of the following laser beam in the dual-laser modes, the main approach is to eliminate the direct effects such as the melt pool boundaries of the remelted zones by the first laser during the following layer. Thus, a comparable material structure for all examined process conditions regarding the resulting melt pool patterns should be obtainable.

The parameter windows for single-laser exposure strategies are mainly described in the literature by laser power P_laser1_, focal spot diameter d_laser1_, scanning speed v_scan_, hatch distance d_hatch_ and layer thickness d_layer_. For expansion of these well-examined parameter windows, the second and following laser beam bring in additional degrees of freedom for adjusting the process conditions. As additional parameters in this study regarding the second laser beam, the laser power P_laser2_, the focal spot diameter d_laser2_ and the offset distance between the laser beams ∆_offset_ were used.

**Figure 6:**
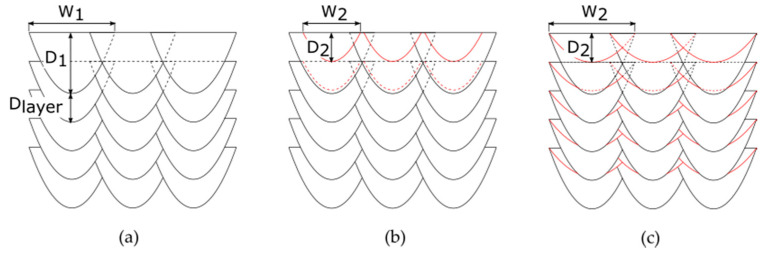
Expected structure with resulting width and depth of the melt pools related to laser 1 (W_1_ and D_1_) and to laser 2 (W_2_ and D_2_) for (**a**) single-laser, (**b**) dual-laser with P_laser2_ of 125 W and d_laser2_ of around 85 µm and (**c**) dual-laser with P_laser2_ of 200 W and d_laser2_ of around 170 µm.

Based on preliminary single-bead studies on FeNiCoMoVTiAl bulk material to derive optimized parameter sets for the PBF-LB processing, resulting melt pool dimensions for different process parameter sets were obtained. Due to the uniform exposure strategy, a sufficient overlapping of the melt pools for the first laser is necessary to reduce the occurrence probability of a lack of fusion defects according to Figure 6a. A good fitting parameter set was identified with P_laser1_ of 200 W, d_laser1_ of approximately 85 µm, v_scan_ of 800 mm/s, d_hatch_ of 0.85 mm and d_layer_ of 40 µm. The resulting melt pool based on the single-bead studies was characterized by a melt pool width W_1_ of around 85 µm and a melt pool depth D_1_ of around 120 µm. Furthermore, two different process parameters for the post-heating laser beam were identified to obtain a melt pool depth each of around 40 µm, which corresponded to the layer thickness. The first parameter set was defined with P_laser2_ of 125 W and d_laser2_ of around 85 µm, as shown in Figure 6b. The second dual-laser parameter set used the possibility to defocus the laser beam to d_laser2_ of around 170 µm by the SCANLAB varioSCAN system to meet the melt pool depth limit of 40 µm despite an increased P_laser2_ of 200 W, as shown in Figure 6c.

The summarized process parameter variations for the dual-laser parameter study are shown in Table 3. The influence of the subsequent laser beam will be investigated in an offset distance range from 1 mm to 4 mm. For ∆_offset_ below 1 mm, strong negative effects on the process stability with delamination issues on the surface layer occurred during preliminary tests. A ∆_offset_ of 2400 mm for DLR-125 and DLR-200 resulted from a 3 s time delay until the surface layer was remelted. In addition to the already defined process conditions, the dimensions of the volumetric test samples were specified to be 10 mm along the scanning vectors, 7 mm wide and around 8 mm high. The build plate preheating was turned off to exclude any influence by a potentially inhomogeneous heat distribution across the build plate surface. Furthermore, the time difference between the exposure processes of two successive layers was set to be 40 s, consisting of 30 s minimum exposure time and 10 s duration for powder recoating.

### 2.6. Evaluation Method

**Figure 7:**
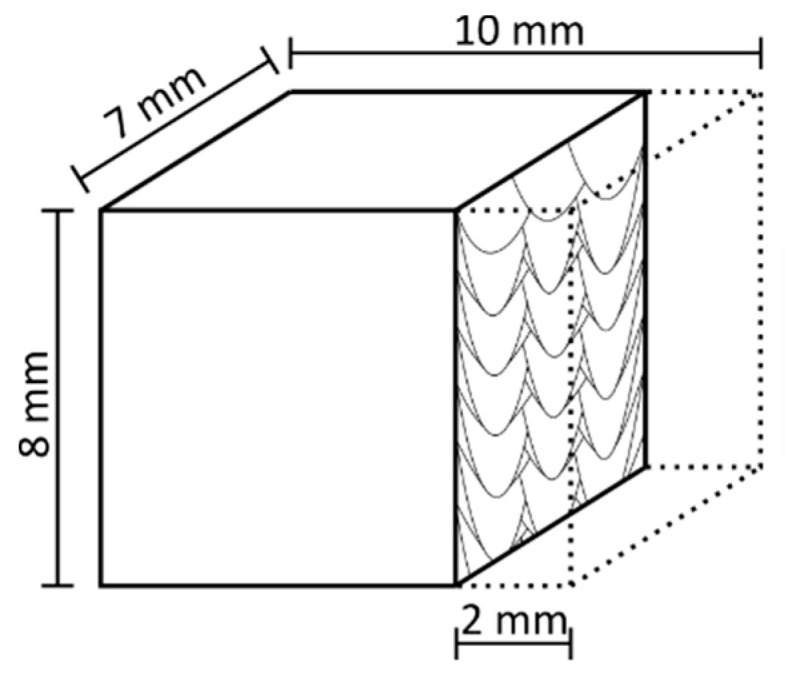
Illustration of resulting cubic test specimen and the preparation plane for further examinations of material density, melt pool structure and hardness.

The plane for evaluation of the produced test samples was aligned, according to Figure 7, vertically to the scanning direction, with an offset of 2 mm to the as-build surface to eliminate edge effects. The cross-sections were ground, polished and analyzed regarding the material density three times in one layer by optical analysis with a Stemi 508 doc stereomicroscope by Carl Zeiss Industrielle Messtechnik GmbH (Oberkochen, Germany) and a 50× magnification. The area of the pores and cracks identified via a constant threshold of the gray value was related to the total area to determine the material density. For the measurements of the resulting melt pool dimensions in the top layer, the cross-sections were etched for 10–20 s with V2A etchant until the surface tarnished. Afterwards, the analysis was performed with the same microscope and a 500× magnification. The width and the depth of the melt pools for laser 1 and laser 2 were determined with 9 measurements each on different melt pools.

For microhardness testing within the melt pool centers from the surface layer downwards, a Q30A+ by ATM Qness GmbH (Mammelzen, Germany) was used. The Vickers hardness impressions were conducted for 10 s to maintain HV0.1 according to DIN EN ISO 6507-1:2018-07 [32]. For standard-compliant measurements, the distances between the roughly 65 hardness indentations were set to every second layer, which resulted in approximately 80 µm each.

## 3. Results and Discussion

### 3.1. Effect on Material Density

**Figure 8:**
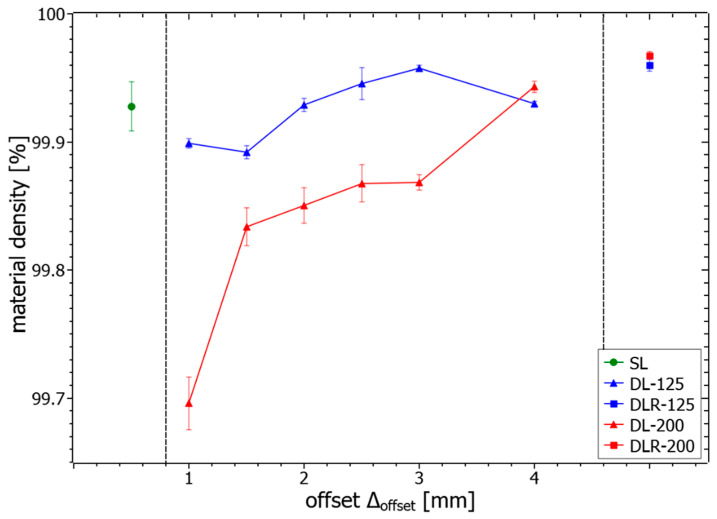
Measured material density values of the samples produced using the listed parameters.

**Figure 9:**
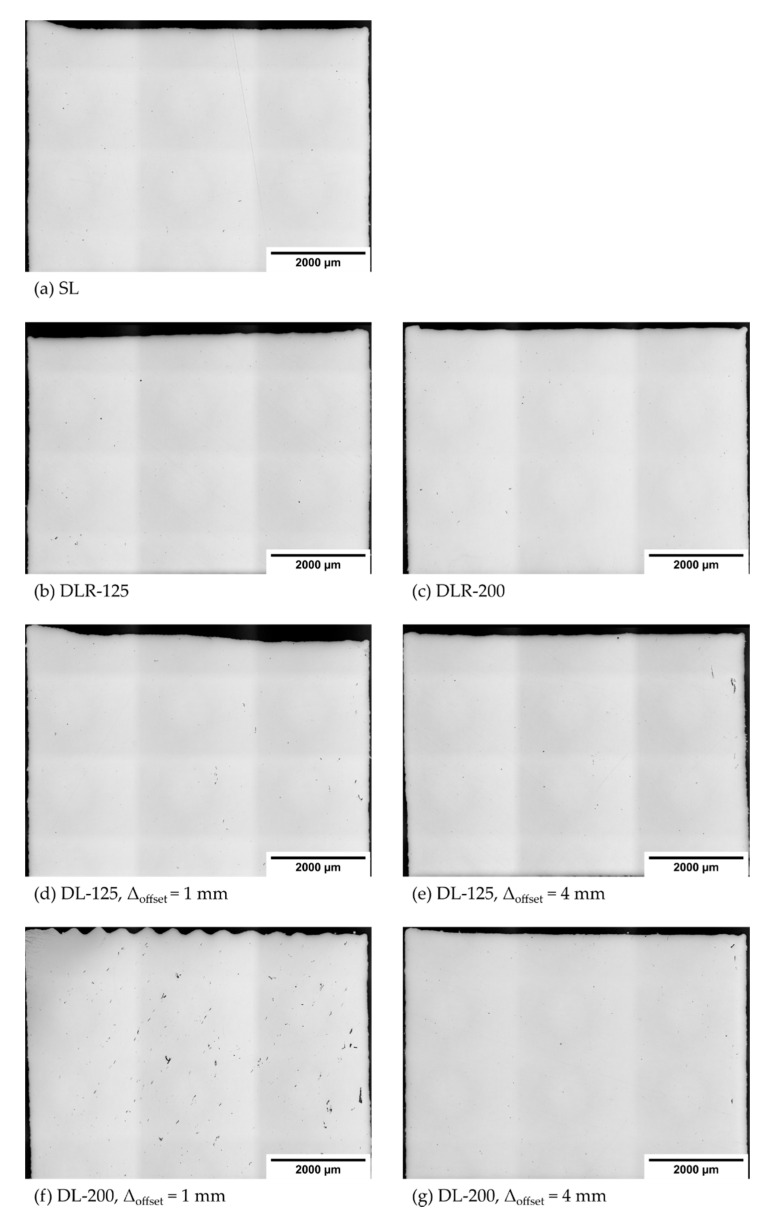
Resulting cross-sections for material density measurements of the parameter sets (**a**) SL, (**b**) DLR-125, (**c**) DLR-200, (**d**) DL-125 with ∆_offset_ of 1 mm, (**e**) DL-125 with ∆_offset_ of 4 mm (**f**) DL-200 with ∆_offset_ of 1 mm and (**g**) DL-200 with ∆_offset_ of 4 mm.

The measured material density values of the different process parameter sets are illustrated in Figure 8. The results show a good overall material density of more than 99.69% for all analyzed samples. In particular, the reference parameter sets SL, DLR-125 and DLR-200 shown in Figure 9a–c have a very high density of more than 99.92%, concluding stable process conditions for the sample manufacturing. There is even a slight material density improvement noticeable on a high level by remelting and thereby eliminating small accumulations on the weld tracks, which could lead to poorer powder deposition on the following layer. The material density determined for DL-125 is nearly constant on a high level of more than 99.89% for all examined offset distances. In comparison, the drop in the material density for DL-200 and ∆_offset_ between 1 mm and 3 mm below 99.90% could be explained by more unstable process conditions due to the instant remelting process with a defocused 200 W powered laser beam. The second laser affects the topography of the surface layer by elevated weld tracks resulting from a higher temperature level and thus the following powder layer deposition as well as melting processes. Due to the layerwise repeated exposure strategies with identical scanning vectors, a buildup of unstable process conditions could occur. The resulting effect could be seen in Figure 9f, with noticeable surface-layer waviness and the tendency to have a higher porosity with larger voids for smaller ∆_offset_ due to the higher amount of laser energy applied in the given time period. This explanation could be supported by the difference in material density between DL-125 with 99.90% and DL-200 with 99.70% for ∆_offset_ of 1 mm. With 200 W laser power, the energy input is around 60% higher in just 1.25 ms after the melting process of the first laser. For ∆_offset_ of 4 mm, also the DLR-200 shown in Figure 9g leads to a comparably high material density of 99.94% due to the longer cooling time between the two laser energy inputs.

### 3.2. Effect on Melt Pool Dimensions

**Figure 10:**
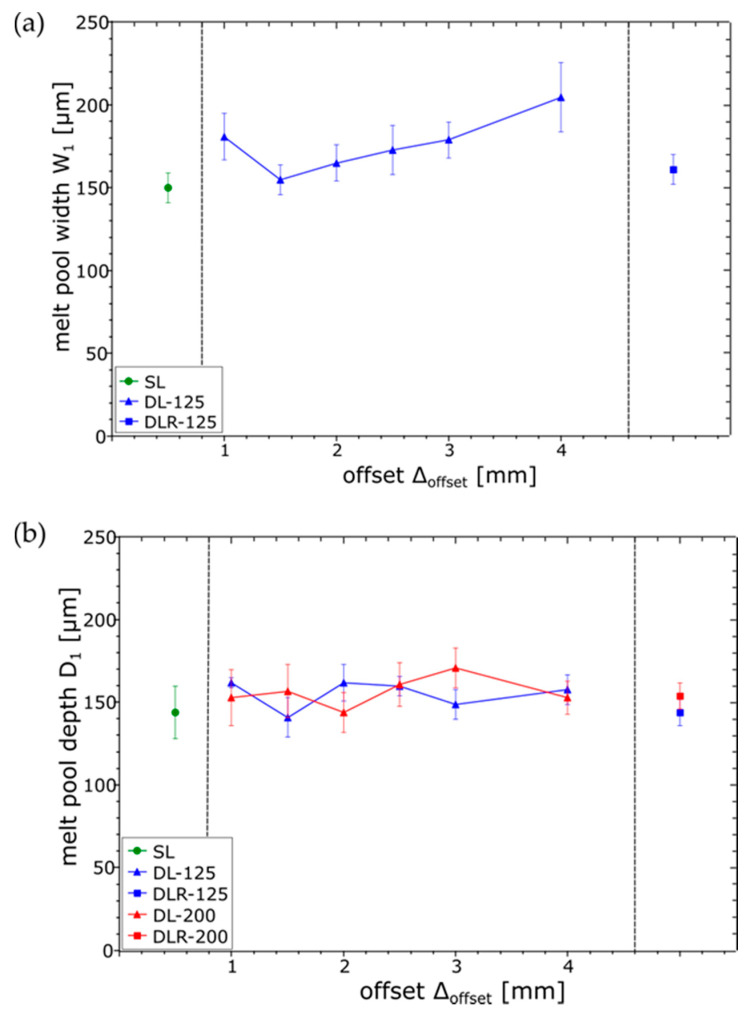
Measured melt pool dimensions ((**a**) width W_1_ and (**b**) depth D_1_) in top layer resulting from laser 1 for the examined process parameter sets.

For obtaining a better understanding of the melting and cooling behavior during the dual-laser PBF-LB processing, the resulting melt pool dimensions of the etched cross-sections were measured in the top layer. Width W_1_ and depth D_1_ of the melt pools are shown in Figure 10 for SL, DL-125 and DLR-125. For DL-200 and DLR-200, only D_1_ could be measured because of the overlapping melt pools of laser 2. As a reference for the melt pool dimensions of laser 1, the parameter set SL can be used. The measured values for D_1_ of 144 µm and W_1_ of 150 µm show a significant increase in the melt pool size compared to the preliminary single-bead studies. This increase could be explained by the higher temperature level of the part during the layerwise manufacturing process and therefore the lower energy input needed for reaching the melting temperature. While D_1_ is almost constant for all process parameter sets as expected, W_1_ shows some irregularities. The increased W_1_ for DL-125 and ∆_offset_ of 4 mm could be explained by the approach of laser 1 to laser 2 on the previous scanning vector. Therefore, a higher temperature level on the surface is expected, which leads to an expansion of the upper region of the melt pool.

**Figure 11:**
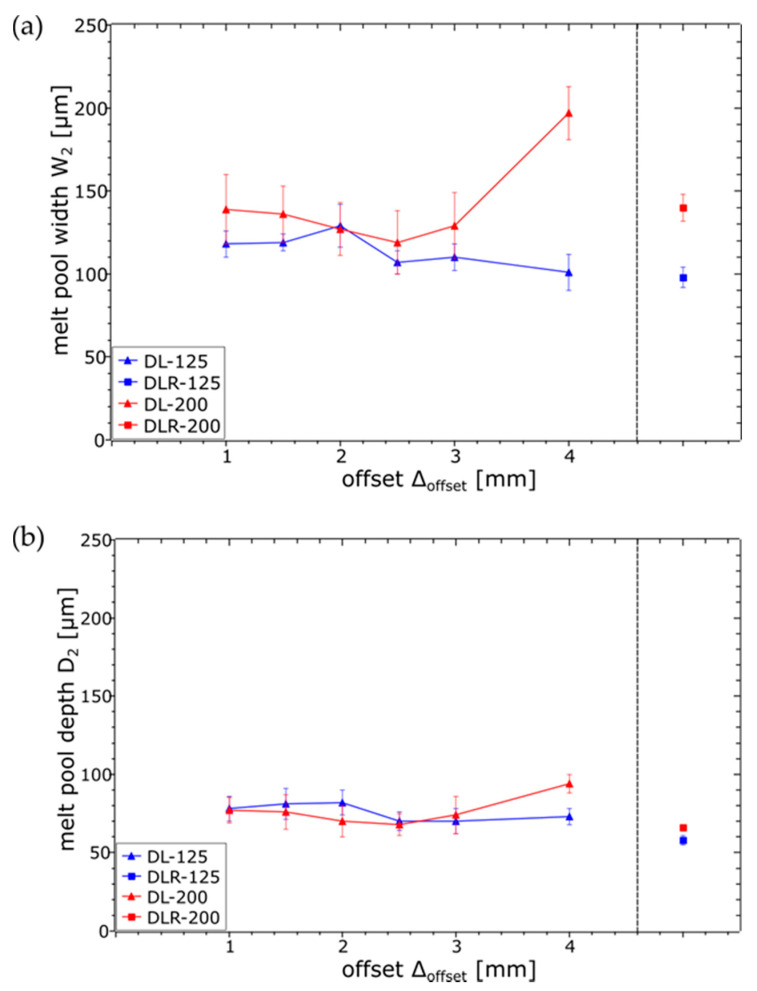
Measured melt pool dimensions ((**a**) width W_2_ and (**b**) depth D_2_) in top layer resulting from laser 2 for the examined process parameter sets.

When it comes to the analysis of the melt pool dimensions for laser 2, shown in Figure 11, the influence of the defocused but higher-power parameter set DL-200 could be clearly seen in the increased W_2_ compared to DL-125, especially for ∆_offset_ of 4 mm. For smaller ∆_offset_ of 1 mm to 3 mm, W_2_ was more difficult to measure for DL-200 because of the already described surface-layer waviness resulting from more unstable process conditions. For DL-125, a slight trend could be obtained for a decreased W_2_ as ∆_offset_ increases due to the longer cooling time between the laser energy inputs. In contrast to W_2_, the D_2_ values for DL-200 and DL-125 are nearly constant, resulting from a constant sub-surface temperature based on the laser 1 energy input and slower heat dissipation within the material.

**Figure 12:**
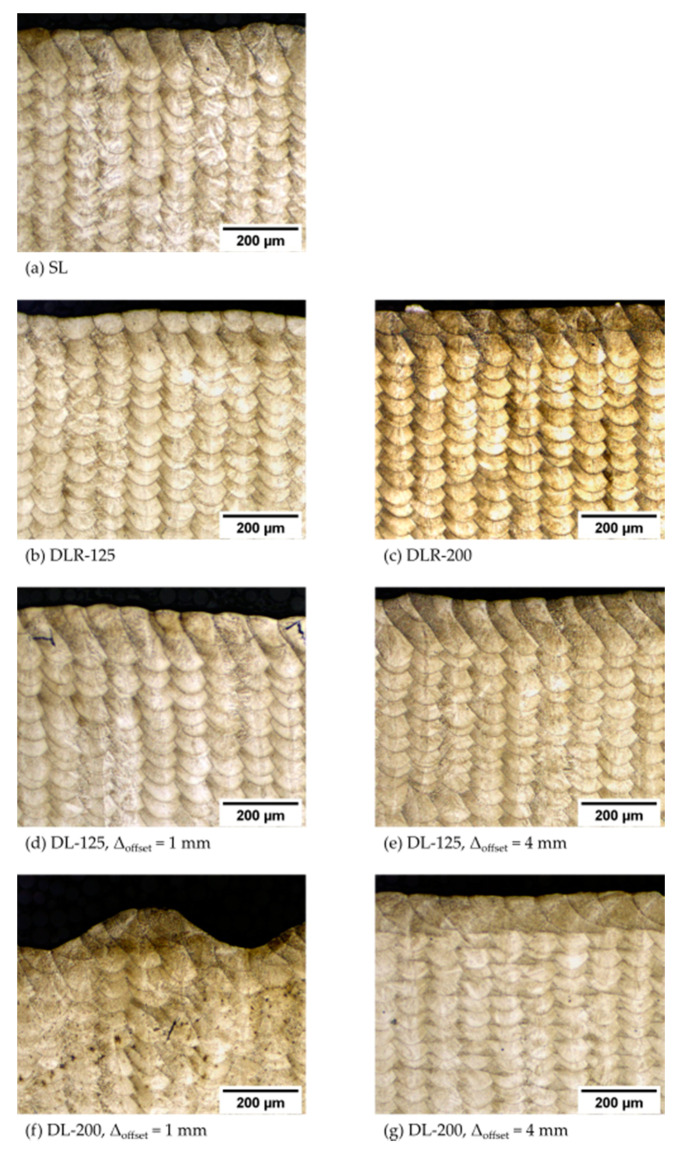
Melt pool cross-sections for measurements of the melt pool dimensions and material structure analysis of the parameter sets (**a**) SL, (**b**) DLR-125, (**c**) DLR-200, (**d**) DL-125 with ∆_offset_ of 1 mm, (**e**) DL-125 with ∆_offset_ of 4 mm (**f**) DL-200 with ∆_offset_ of 1 mm and (**g**) DL-200 with ∆_offset_ of 4 mm.

When analyzing the etched cross-sections in Figure 12, only minor differences between the melt pool structures of the different parameter sets could be identified. Most of the resulting material structure was apparently only melted by laser 1 and only differed in the thermal history during the layerwise dual-laser PBF-LB processing. This shows a good implementation of the originally intended dual-laser exposure strategy, even though the resulting melt pool dimensions increased compared to the originally intended dimensions due to the higher energy input and associated temperature level of the part during PBF-LB processing.

### 3.3. Effect on Micro Hardness

**Figure 13:**
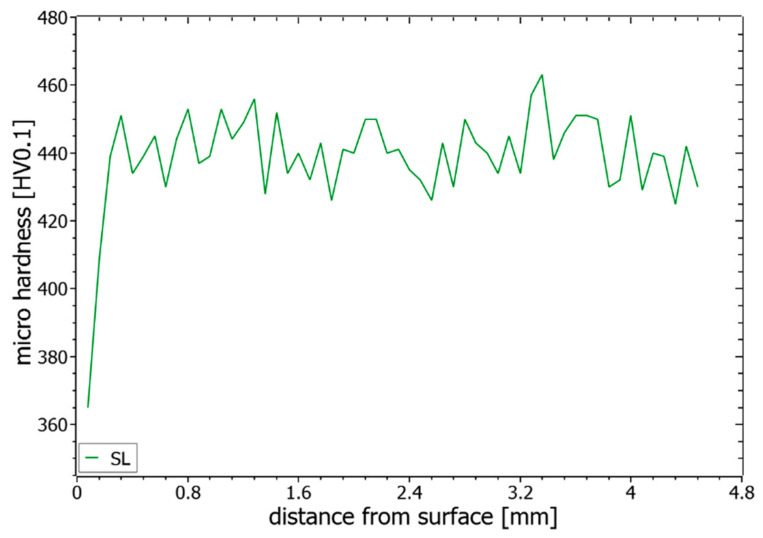
Exemplary microhardness profile over build height of the parameter set SL.

In Figure 13, an exemplary hardness profile is shown for SL, which is typical for all evaluated hardness results. A significant drop in the hardness within an area of around 400 µm below the surface layer could be seen for all samples until the hardness values ranged around a stable hardness level of 441 HV. The reason for this drop could be seen in the process-related temperature history and the need for multiple heating and cooling cycles on a higher temperature level for receiving the respective hardness. In order not to influence the following evaluations too strongly by the effect near the surface, only measured values below the 400 µm distance are considered.

**Figure 14:**
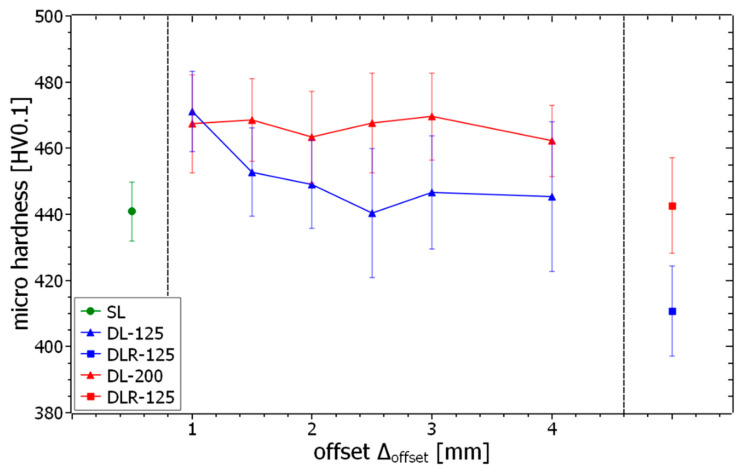
Measured microhardness values of the samples produced with the different process parameter sets.

The mean values and standard deviations for the microhardness examination of the different process parameter sets and offsets are shown in Figure 14. The hardness level of SL, with around 441 HV0.1, can be seen as a reference value for the evaluation of the dual-laser and remelting parameter sets. For the dual-laser processing with offset distances between 1 mm and 4 mm, the results show a significant trend to a higher microhardness, with values up to 471 HV0.1 for DL-125 and DL-200. Although DL-125 sets the overall maximum hardness with ∆_offset_ of 1 mm, the DL-200 parameters show a more constant hardness level between 462 HV and 470 HV. Due to the microhardness testing, the slightly lower porosity of the DL-200 parameter sets below ∆_offset_ of 4 mm has no detectable influence on the hardness. For ∆_offset_ from 1.5 mm to 4 mm, the hardness level for DL-125 drops down to a minimum value of 440 HV, with an almost continuous progression. The assumption is thus that the energy input for DL-125 is only sufficient to trigger the significant hardness increase effects such as short-term aging in the material for very short ∆_offset_ values below approximately 1.5 mm. In the case of DL-200, this more stable hardness increase effect can be explained by the higher energy input and the associated longer holding time at a higher temperature level in the in-situ heat treatment zone. Due to the slower heat dissipation processes below the surface, the DL-200 parameter sets appear to be more independent of variation of ∆_offset_. The examination of the remelting parameter sets shows DLR-200 at a comparable hardness level with SL. For DLR-125, the hardness significantly decreases below the hardness of SL to around 411 HV0.1. Obviously, there is a material-specific effect discovered for ∆_offset_ between 4 mm and 2400 mm that leads to a decreased hardness. Possibly, a solution annealing process is started due to the time-delayed reheating of the in-situ heat treatment zones leading to this effect.

All these detected changes in hardness levels for the different process conditions could be related to material-specific effects based on the different temperature histories during the manufacturing process. Shorter distances and higher energy inputs of the subsequent laser beam lead to harder materials and remelting parameters lead to softer material properties compared to standard single-laser process parameters.

### 3.4. Observed Potential for Functionally Graded Materials by Double-Laser PBF-LB Processing

To reach the full potential of voxel-based manufacturing in PBF-LB, applicable processing strategies and suitable materials must be qualified. The needed PBF-LB systems for using multiple laser beams at least in a certain overlapping area are already commercially available and the further strategy of the system OEMs already tends towards larger systems with more lasers. The identified changes in hardness related to different process parameter sets and the resulting thermal histories could be used for an intentionally graded modification in the mechanical properties of additively manufactured parts. Due to the consistent melt pool structure for all considered parameter sets, no negative effects are assumed if the parameter sets are changed on certain regions during part production. This could be one of the main advantages for the specific adjustment of the in-situ heat treatment parameters with a subsequent laser beam compared to modified single-laser parameters with significant changes in melt pool geometries during part manufacturing. In particular, when using a material for tooling applications, such as the newly developed maraging FeNiCoMoVTiAl alloy, a functionally graded material structure could be used to produce parts with hard and wear-resistant outer surfaces in conjunction with ductile core regions. As the hardness changes are derived from material-specific effects due to the different thermal histories while processing, there is furthermore a chance of maintaining these graded properties even through a subsequent aging heat treatment process of the part. However, a further solution heat treatment will eliminate the discovered hardness changes by dissolving the characteristic PBF-LB microstructure.

## 4. Conclusions

The results show the feasibility of dual-laser PBF-LB processing of the maraging tool steel FeNiCoMoVTiAl by using a second laser beam for post-heating and remelting a fraction of the previously welded tracks for the modification of in-situ heat treatment conditions. A sufficient calibration procedure and minor hardware modifications are needed to build volumetric parts with a uniform exposure strategy based on a G-code-like vector dataset. Furthermore, the main results for the different evaluation methods are listed below:

1. A material density of more than 99.69% was obtained for all tested parameter sets. The best density results were determined for single-laser and remelting parameters with more than 99.92%. The dual-laser parameters with the subsequent 125 W laser beam resulted in a material density of more than 99.89% due to the more stable processing conditions in comparison to the dual-laser parameters with 200 W.
2. Significant changes in the resulting melt pool dimensions regarding the parameter variations of the subsequent laser beam were measured on the surface layer. As a result of the elaborated exposure strategies, a consistent melt pool structure was still obtainable for all tested parameter sets.
3. The modified dual-laser process parameter sets led to a significant hardness change compared to single-laser PBF-LB processing with around 441 HV0.1. An increase by around 30 HV0.1 was obtained by using the dual-laser exposure strategies. A higher laser power as well as a shorter offset distance of the subsequent laser beam appear to have hardness-increasing effects on the resulting material structure. Possibly, short-time aging effects of the in-situ heat treatment zone could be the reason for the hardness increase. A hardness decrease by around 30 HV0.1 compared to the single laser parameter was determined by a remelting strategy with 125 W laser power. Solution annealing effects within the heat-affected zone could be a possible explanation for this.
4. The adjustable hardness levels for the different dual-laser parameter sets in combination with no significant changes in the resulting melt pool structure open new possibilities to produce functionally graded material structures by dual-laser PBF-LB processing.

Further research will be conducted regarding the necessary heat treatment after dual-laser PBF-LB processing to receive the material-specific properties of maraging tool steels. Ideally, a heat treatment process can be identified that also preserves the functionally graded effects when several processing conditions are combined in one component. This would enable the pursuit of industrial application of the results.

## Figures and Tables

**Table 1:** Chemical composition of the maraging tool steel Specialis^®^.

Element	Fe	C	Ni	Co	Mo	Ti	V	Al
wt%	Bal.	0.02	18.33	11.39	4.44	<2	<2	<0.5

**Table 2:** Powder specification according to VDI 3405 Blatt 2:2013-08.

Powder Parameter		Values	Measurement Method
Particle size distribution	d_10,3_ (µm)	19.32	Dynamic particle imaging with Camsizer X2 by Retsch GmbH (Haan, Germany)
	d_50,3_ (µm)	32.30	
	d_90,3_ (µm)	54.91	
Particle sphericity	SPHT3	0.85	
Flowability	(s/50 g)	14.86	Hall flowmeter according to DIN EN ISO 4490:2018 [30]
Residual moisture	(%)	<5	humimeter RH2 by Schaller Messtechnik GmbH (St. Ruprecht an der Raab, Austria)

**Table 3:** Overview of parameter sets for the specimen manufacturing.

Parameter Set	Acronym	P_laser2_ (W)	d_laser2_ (µm)	∆_offset_ (mm)
Reference single-laser	SL	0	-	-
Double-laser 125 W	DL-125	125	85	1, 1.5, 2, 2.5, 3, 4
Reference remelting 125 W	DLR-125	125	85	2400
Double-laser 200 W	DL-200	200	170	1, 1.5, 2, 2.5, 3, 4
Reference remelting 200 W	DLR-200	200	170	2400

## Data Availability

The data presented in this study are available on request from the corresponding author after obtaining permission of authorized person.
